# Etiology and Clinical Presentation of Disorders of Sex Development in Kenyan Children and Adolescents

**DOI:** 10.1155/2019/2985347

**Published:** 2019-12-01

**Authors:** Prisca Amolo, Paul Laigong, Anjumanara Omar, Stenvert Drop

**Affiliations:** ^1^Paediatric Endocrinology Training Centre for Africa, Nairobi, Kenya; ^2^Department of Paediatrics and Child Health, University of Nairobi, Nairobi, Kenya; ^3^Department of Paediatrics, Division of Paediatric Endocrinology, Erasmus MC-Sophia, Rotterdam, Netherlands

## Abstract

**Objective:**

The purpose of this study was to describe baseline data on etiological, clinical, laboratory, and management strategies in Kenyan children and adolescents with Disorders of Sex Development (DSD).

**Methods:**

This retrospective study included patients diagnosed with DSD who presented at ages 0–19 years from January 2008 to December 2015 at the Kenyatta National (KNH) and Gertrude's Children's (GCH) Hospitals. After conducting a search in the data registry, a structured data collection sheet was used for collection of demographic and clinical data. Data analysis involved description of the frequency of occurrence of various variables, such as etiologic diagnoses and patient characteristics.

**Results:**

Data from the records of 71 children and adolescents were reviewed at KNH (*n* = 57, 80.3%) and GCH (*n* = 14, 19.7%). The mean age at the time of diagnosis was 2.7 years with a median of 3 months. Thirty-nine (54.9%) children had karyotype testing done. The median age (IQR) of children with reported karyotypes and those without was 3.3 years (1.3–8.9) and 8.3 years (3.6–12.1), respectively (*p*=0.021). Based on karyotype analysis, 19 (48.7%) of karyotyped children had 46,XY DSD and 18 (46.2%) had 46,XX DSD. There were two (5.1%) children with sex chromosome DSD. Among the 71 patients, the most common presumed causes of DSD were ovotesticular DSD (14.1%) and CAH (11.3%). Majority (95.7%) of the patients presented with symptoms of DSD at birth. The most common presenting symptom was ambiguous genitalia, which was present in 66 (93.0%) patients either in isolation or in association with other symptoms. An ambiguous genitalia was initially observed by the patient's mother in 51.6% of 62 cases despite the high rate (84.7%) of delivery in hospital. Seventeen (23.9%) of the cases had a gender reassignment at final diagnosis. A psychologist/psychiatrist or counselor was involved in the management of 23.9% of the patients.

**Conclusion:**

The commonest presumed cause of DSD was ovotesticular DSD in contrast to western studies, which found CAH to be more common. Investigation of DSD cases is expensive and needs to be supported. We would have liked to do molecular genetic analysis outside the country but financial challenges made it impossible. A network for detailed diagnostics in resource-limited countries would be highly desirable. There is a need to train health care workers and medical students for early diagnosis. Psychological evaluation should be carried out for all patients at diagnosis and support given for families.

## 1. Introduction

Disorders of Sex Development (DSD) are congenital conditions with atypical development of chromosomal, gonadal, or anatomical sex [1]. The classification of ambiguous genitalia in patients is difficult because similar phenotypes may have several etiologies [2–6]. In 2006, Lawson Wilkins Pediatric Endocrine Society (LWPES) and European Society for Paediatric Endocrinology (ESPE) proposed a classification system for causes of intersex disorders on the basis of karyotype analysis [4]. This classification includes three diagnostic categories: sex chromosome DSD, 46,XY DSD (formerly male pseudohermaphroditism) and 46,XX DSD (formerly female pseudohermaphroditism).

There is a paucity of data on the incidence of DSD; it is estimated that the overall incidence of DSD is one in 5,500 [7, 8]. Congenital adrenal hyperplasia (CAH) and mixed gonadal dysgenesis (MGD) are the most common causes of ambiguous genitalia, constituting approximately over 50% of all cases of genital ambiguity in the newborn period [9]. The incidence of CAH and MGD worldwide is 1 : 15,000 and 1 : 10,000, respectively, but varies considerably among different populations [10–12].

In Kenya, there are currently no data on the frequency of etiological diagnoses and clinical presentation of DSD in Kenyan children and adolescents. Furthermore, diagnosis and management of DSD in resource-limited settings like ours represent a major challenge. Awareness among health care workers is thought to be poor resulting in delayed diagnosis and high morbidity and mortality.

The aim of this study was to describe the baseline data on etiological distribution, clinical characteristics, laboratory evaluation, and management strategies in Kenyan children and adolescents with DSD. Due to the challenges of conducting molecular genetic analysis in our setting, the diagnosis of DSD in this study was based on clinical and biochemical features. We hope that the study results will be used to inform future policies that will enhance the quality of care given to these patients as well as add to the body of knowledge on DSD from this region. It will also serve as a basis for appropriate further research.

## 2. Methods

We retrospectively evaluated in January and February 2016 the records of 71 consecutive patients on follow-up during the 8-year period between January 2008 and December 2015 at the Departments of Paediatrics and Surgery at Kenyatta National Hospital (KNH) and Gertude's Children's Hospital (GCH) in Nairobi, Kenya.

The study subjects were children who had presented at ages 0–19 years to the above two hospitals during the 8-year study period and were confirmed to have a DSD based on clinical, laboratory, and radiological evaluation and/or surgical laparotomy/laparoscopy. Patients with noncongenital (acquired) problems of late puberty were excluded from the study.

A written approval was obtained from each of the study hospitals before collection of data in the respective hospitals. Clinical information on age at presentation, complaints, gender of rearing, consanguinity, family history of genital ambiguity or fetal/infant deaths, anthropometry, assessment of pubertal stage, presence of hyperpigmentation, hypertension, associated anomalies, or dysmorphic features was obtained from patient records and recorded on the data collection sheet. We also obtained information on sociodemographic data, specifically relating to age, level of education of both the patient, and the parent/guardian and whether in paid employment or not (parent/guardian). We used the Prader Scoring System and External Masculinization Score (EMS) to determine the degree of external virilization [13]. Criteria that suggested DSD included overt genital ambiguity, apparent female genitalia with clitoromegaly, posterior labial fusion or inguinal/labial mass, apparent male genitalia with nonpalpable testes, micropenis, isolated perineal or penoscrotal hypospadias, mild hypospadias with undescended testis, and delayed puberty. We obtained information from the records on the investigations done including karyotype analysis, hormone measurements, abdominopelvic imaging, laparotomy/laparoscopy, and biopsy. We also searched for patients with Turner Syndrome and Klinefelter Syndrome which are included in the DSD classification.

The subjects who had a karyotype analysis were classified into three etiologic groups: 46,XY DSD, 46,XX DSD, and sex chromosome DSD. A normal baseline testosterone level (above 1 nmol/L) or a three-fold increment in plasma testosterone after human chorionic gonadotropin (hCG) stimulation was considered to indicate the presence of functioning testicular tissue [13, 14]. A testosterone (T) to dihydrotestosterone (DHT) ratio greater than 12 was accepted as suggestive of 5*α*-reductase deficiency [15]. Androgen insensitivity syndrome (AIS) was presumed in individuals with a 46,XY karyotype who had normal T and DHT response (three-fold increment) to hCG stimulation and absence of müllerian structures. The presence of normal female external genitalia was considered suggestive of complete androgen insensitivity syndrome (CAIS), while the rest were considered as presumptive partial androgen insensitivity syndrome (PAIS). Gonadal regression (vanishing testes syndrome) was presumed in patients with normal male external genitalia, low basal (below 1 nmol/L) or no testosterone response to hCG, and bilateral anorchia on imaging.

A presumptive diagnosis of 21-hydroxylase deficiency was made in individuals with müllerian structures and elevated basal 17-hydroxyprogesterone (17-OHP) (above 3 nmol/L, based on in-house laboratory cutoff values) [16]. Gonadal dysgenesis was presumed in patients who had underdeveloped/absent gonads detected by imaging studies or surgical exploration. The diagnosis of ovotesticular DSD was based on surgical exploration and gonadal biopsy [17].

For each individual with missing measurements at first presentation to the health facility such as height, weight, and blood pressure, the missing value was replaced by the next observed value of that variable (next observation carried backward (NOCB)). The maximum period for imputing the value in the next visit was two months. Any variable that was not available for 80% or more of the study sample was not included in the analysis. Individual cases that had more than 80% missing data were omitted from the study (available case analysis). We discarded two patients with records that had more than 80% missing data, one with suspected Klinefelter syndrome and another with suspected Turner syndrome.

Data were analyzed accordingly using Statistical Product and Service Solutions (SPSS) version 17. Chi-squared tests were used to compare categorical variables and proportions across groups. Continuous variables were compared using student's *t* test and Mann–Whitney *U* test. A *p* value of less than 0.05 was considered statistically significant.

### 2.1. Ethical Consideration

The study was conducted after obtaining written approval from the Kenyatta National Hospital Ethics and Research Committee and Gertrude's Children's Hospital Ethical Review Board in Kenya.

## 3. Results

### 3.1. Age

**Figure 1:**
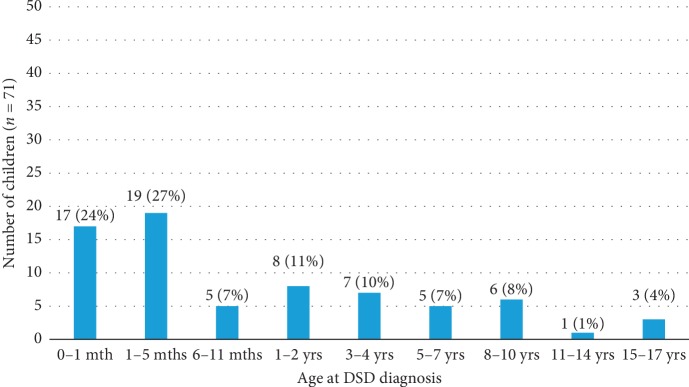
Age at diagnosis.

The age at the time of diagnosis among the 71 patients varied widely from birth to 17 years. The mean age was 2.7 years and median age 3 months, with the majority (51%) of the children aged 5 months and below (Figure 1).

### 3.2. Clinical Presentation

Majority (95.7%) of the patients presented with symptoms of DSD at birth. The most common presenting symptom was ambiguous genitalia, which was present in 66 (93.0%) patients either in isolation or in association with other symptoms.

Six patients with ambiguous genitalia had syndromic features/multiple congenital malformations. One of these patients who was diagnosed with Edward syndrome had microcephaly, a low hairline and low-set ears, and had been exposed to prenatal progestagens due to a threatened abortion. Another patient had hirsutism and low-set ears. The third patient had congenital talipes equinovarus, tongue-tie, lip-tie, obesity, and renal malformations with recurrent urinary tract infections. This patient had been exposed to aspirin throughout pregnancy for maternal chronic hypertension. The fourth patient presented with anorectal malformation and congenital talipes equinovarus, while the fifth one had facial hypertrichosis, a short neck and a small chin. One neonate who was HIV-exposed presented with ambiguous genitalia and spina bifida cystica.

Two patients presented with ambiguous genitalia with acne and development of pubic hair at 3 months and 5.5 years respectively. One patient with ambiguous genitalia had salt-wasting crisis at presentation while another had bilateral inguinal herniae.

### 3.3. Anthropometric Measurements

#### 3.3.1. Weight and Height

The findings of physical measurements based on the Centers for Disease Control and Prevention (CDC) growth standards showed that 25 participants (39.7% of reported measurements) had weights for age below −2SD at first visit, and 20 participants (44.4% of reported measurements) had height/length for age below −2SD. Sixteen percent of these children had been born prematurely. Over half (53%) of these patients were from the capital city of Nairobi while the rest had been referred from outside Nairobi.

#### 3.3.2. Blood Pressure

Out of 16 children who had blood pressure measurements recorded, 12 (75%) were normotensive and 4 (25%) were hypertensive. Among the hypertensive patients, one was syndromic with anorectal malformation and congenital talipes equinovarus. Gonadal dysgenesis with renal failure based on a WT1 mutation is possible in this patient who had a 46,XY karyotype and was yet to undergo a diagnostic laparotomy/laparoscopy. Another patient had sex chromosome DSD (Turner variant 48,XYYY/45,X). This patient had been referred for a karyotype and was transferred back to the referring hospital for follow-up. One patient had a presumed disorder of testosterone biosynthesis while another did not have the diagnosis established due to loss to follow-up. Among the eight patients with presumed CAH, four had blood pressure readings documented and all were normal.

### 3.4. Family History and Pregnancy-Related Factors

#### 3.4.1. Genital Ambiguity

Among 58 patients, 6 (10.3%) reported a family history of genital ambiguity. Two of these patients had a 46,XY karyotype and had a presumed disorder of testosterone biosynthesis. One had a maternal cousin who was initially female and later changed to a male while the other had a history of hypospadias in a first-born sibling. One patient with 46,XX ovotesticular DSD had a history of micropenis in an unspecified family member. A patient with 46,XX testicular DSD had a cousin with a history of undescended testes. Two patients who did not have an established diagnosis had a history of micropenis in a maternal uncle and an uncle with ambiguous genitalia, respectively. Family history of genital ambiguity was not known in 13 patients.

#### 3.4.2. Infertility

Five (15.2%) patients out of 33 had a family history of infertility. All these five patients had the history of infertility reported in the paternal aunt. The family history of infertility was not known in 38 patients.

#### 3.4.3. Fetal/Infant Loss

There was a family history of fetal loss/infant deaths reported in nine (30%) out of 30 cases. Family history of fetal/infant loss was not known in 41 cases. One patient with CAH had a history of death in a sister at 10 months of age, with the cause of death reported as “malaria” by the parent. Two patients with CAH did not have a history of fetal/infant death while the remaining five had no documentation on the same.

#### 3.4.4. Consanguinity

A history of parental consanguinity was reported in three (12.5%) out of 24 cases. In two of these cases, the parents were reported to be from the same clan, while in the third case, the exact relationship of the parents was not documented. History of parental consanguinity was not known in 47 cases.

#### 3.4.5. Antenatal Drug Exposure

Among 60 patients, 2 (3.3%) had been exposed to drugs during the antenatal period. The first patient who was diagnosed with Edward syndrome (47,XX, +18) had been exposed to progestagens in the first trimester for a threatened abortion and has been described above. In the second case also described above, the mother was on aspirin throughout the pregnancy for chronic hypertension. This child had a 46,XX karyotype. The history of antenatal drug exposure was not known in 11 patients.

### 3.5. Initial Management

Fifty patients were delivered in hospital and nine at home. There was no documentation on place of delivery for 12 patients.

Ambiguous genitalia was initially observed by the patient's mother in 32 cases and by a health professional in 28 cases. It was not clear from the documentation who initially observed the genital ambiguity in nine patients.

Thirty-six (50.7%) patients were reared as male while 31 (43.7%) had been raised as females. The remaining four patients had not been assigned a gender from birth. Among those who had karyotypes done, 19 patients had a genotype that was discordant with the gender of rearing (10 males with 46,XX karyotype, and 9 females with 46,XY karyotype).

### 3.6. Diagnoses

**Figure 2:**
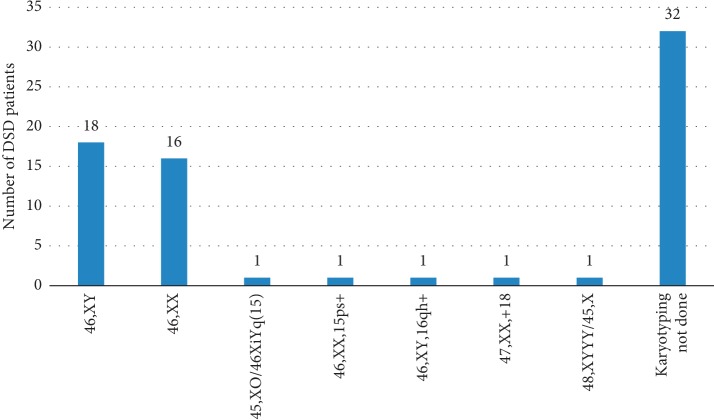
Karyotypes.

Out of the 71 children in the sample, 39 (54.9%) had karyotype testing done (Figure 2). The rest did not have karyotype testing due to economic reasons. The median age (IQR) of children with reported karyotypes and those without was 3.3 years (1.3–8.9) and 8.3 years (3.6–12.1), respectively, (*p*=0.021).

Based on karyotype analysis, 19 (48.7%) of karyotyped children had 46,XY DSD and 18 (46.2%) had 46,XX DSD (Table 1). There were two (5.1%) children with sex chromosome DSD.

Among all the 71 patients, 10 (14.1%) had a presumed diagnosis of ovotesticular DSD (Table 2). Out of 8 (11.3%) cases with presumed CAH, 5 had the simple virilizing form, 2 nonclassic 21-hydroxylase deficiency, and 1 salt-wasting 21-hydroxylase deficiency. All of these patients with presumed CAH except 2 had a basal 17-OHP level ranging from 39.6 to 1050 nmol/L. One patient who had presented with salt-wasting adrenal crisis at 1 month of age and was on hydrocortisone and table salt at the time of testing had a 17-OHP of 6.7 nmol/L. The second one who had presented with ambiguous genitalia had a 17-OHP of 3.6 nmol/L, a 46,XX karyotype, elevated testosterone levels, and low cortisol level based on in-house laboratory cutoff values.

A presumed diagnosis of 5*α*-reductase deficiency was made in two patients. One patient was presumed to have PAIS, and there was none with CAIS. We were not able to perform mutation analysis of the androgen receptor (AR) gene. Four patients were presumed to have disorders of testosterone biosynthesis. Two of these patients had a 46,XY karyotype and a poor testosterone response to hCG stimulation. The third patient who was aged 14 years and 11 months had a 46,XY karyotype, undetectable basal testosterone (hCG test not done), and right intra-abdominal and left inguinal testes with no Műllerian structures on laparotomy. The fourth patient aged 4 years and 8 months had a 46,XY karyotype, low basal testosterone (hCG test not done), and bilateral intra-abdominal testes with no Műllerian structures on laparotomy.

There were 6 patients with 46,XY karyotype in whom it was not established whether there was a disorder of androgen action or synthesis. Five of these patients had incomplete hCG stimulation tests while one had no biochemical tests done.

Twenty-six (36.6%) patients did not have the etiologic diagnosis established. Ten of these patients were lost to follow-up. The commonest presumed diagnosis among the nonkaryotyped patients was CAH (15.6%) and ovotesticular DSD (12.5%).

### 3.7. Investigations

Forty-nine (69%) patients had an abdominopelvic ultrasound done while 35 (49.3%) had a laparoscopy or laparotomy as part of the investigations. Histology of the gonads was performed for 20 (28.2%) patients. No patient was found to have a gonadal tumour. Four out of the 8 patients with CAH had bone age determined at presentation and 3 of these patients had advanced bone age.

### 3.8. Gender Assignment

Thirty-nine (54.9%) patients were finally assigned a male gender, while 16 (22.5%) were assigned a female gender by the multidisciplinary DSD team consisting of an endocrinologist, surgeon, psychologist, radiologist, and social worker. The team took into account the supposed diagnosis and tried to match the gender assignment to the patient's chromosomal and gonadal sex where possible, while trying to anticipate pubertal development and future function and fertility.

The average age for gender assignment was 3.4 years. There was no documented gender incongruence/gender dysphoria. Thirteen (18.3%) of the patients did not have a final gender assigned, nine of whom were lost to follow-up. The remaining four were still undergoing diagnostic and psychiatric evaluation. Information was not available on the final gender assignment for three patients who had been transferred back to the referring facility.

Seventeen (23.9%) of the cases had a gender reassignment at final diagnosis. Among these patients, 76.5% were reassigned from female to male gender while 23.5% were reassigned from male to female gender. Among those reassigned from female to male gender, the presumed diagnoses included 5*α*-reductase deficiency, PAIS, mixed gonadal dysgenesis, and unclassified disorders of androgen synthesis or action. Those reassigned from male to female gender included two patients with CAH and one with 46,XX karyotype in whom the exact etiology was not established. Twelve of these patients were below 4 years and 5 were above 5 years (5 years, 6.5 years, 7 years 1 month, 9 years 5 months, and 15 years 8 months) at the time of reassignment.

### 3.9. Medical and Surgical Treatment

Six of the patients with CAH were on hydrocortisone while one was on prednisolone due to financial constraints. The patient with salt-wasting CAH was also on treatment with table salt.

Three patients with 46,XY DSD and the one with mixed gonadal dysgenesis received topical testosterone as part of the treatment. Twenty-four patients underwent genitoplasty/urethroplasty while 9 patients underwent orchidopexy. Two patients with ovotesticular DSD who were assigned male gender underwent oophorectomy while one with ovotesticular DSD assigned a female gender underwent bilateral gonadectomy.

### 3.10. Specialists Involved in Care of Patients

A paediatric surgeon was involved in the management of 78.9% of the patients and a paediatric endocrinologist in 77.5% of the cases. A psychologist/psychiatrist or counselor was involved in the management of 23.9% of the patients. Among the 28 patients who underwent psychosocial evaluation, 20 had psychosocial issues such as aggressive behavior, poor school performance, anxiety, withdrawal, and eating disorder.

### 3.11. Follow-Up Outcome

In terms of outcome, 50.7% of patients were still being followed up in the clinics while 36.6% were lost to followup. Some patients (11.3%) were transferred back to the referring facilities for follow-up, while for 1.4%, there was inadequate documentation to determine the outcome. As shown in Table 3, karyotyped patients were more likely to be still on follow-up at the clinics compared to nonkaryotyped patients (*p* value < 0.001).

## 4. Discussion

According to the 2018 Report of the Taskforce on Policy, Legal, Institutional, and Administrative Reforms regarding the Intersex Persons in Kenya [18], only 5% of persons with DSD recognize themselves as intersex, while the others are mostly confused about their exact status due to lack of awareness and support. In terms of legal recognition and documentation, the taskforce found that majority of persons with DSD had birth certificates, but the recorded gender conflicted with the assigned gender [18]. The birth certificates make it difficult to acquire identity cards with resultant negative social implications such as missed employment and voting opportunities [18].

In this retrospective study of 71 youth with DSD at Kenyatta National and Gertrude's Children's Hospitals in Nairobi, the most common presumed causes of DSD were ovotesticular DSD (14.1%) and CAH (11.3%). In contrast, in South Africa, Ganie et al. [19] found the most common diagnoses to be unclassified disorder of androgen synthesis and action (53%) and ovotesticular DSD (22%). In Turkey, Erdoğan et al. [20] found that the most common causes of DSD were Turner syndrome and CAH, while Parisi et al. [21] found CAH (14%) and AIS (10%) to be the commonest causes of DSD in Seattle.

Although ovotesticular DSD is rare [17, 22], it is thought to be more prevalent in South African blacks [23], particularly 46,XX ovotesticular DSD. Most cases are sporadic with few documented cases of familial recurrence. There are more than 400 cases reported worldwide [24].

In our study, 6 out of the 10 patients with presumed ovotesticular DSD had a 46,XX karyotype while the other 4 did not have a karyotype done. Krob et al. [23] observed that out of the 96 cases described in Africa, 96.5% showed a 46,XX karyotype.

An ovotestis was the most common gonad found in 58.8% of all gonads, while an ovary was found in 23% and a testis in 17.7%. This finding was similar to other studies [22, 23, 25].

Among our patients, bilateral ovotestes, an ovotestis and ovary, and a testis and ovary were present in 2 patients each, while 2 patients had a right ovotestis and an absent gonad on the left. Ganie et al. [22] found an ovotestis and ovary to be the most common combination of gonads followed by bilateral ovotestes. Gonads with testicular tissue have been found to be more frequent on the right side of the body, while pure ovarian tissue is more common on the left [22, 23]. Krob et al. [23] observed that histologically, ovarian tissue often appeared normal while testicular tissue was described to be immature. This is in agreement with our findings.

In our study, 48.7% of karyotyped children had 46,XY DSD and 46.2% had 46,XX DSD. This is similar to findings in other studies [20, 21, 26, 27]. In contrast, in Indonesia, Juniarto et al. [28] found 46,XY DSD to be even more common, accounting for 63.3% of cases. Our data demonstrate that the commonest presumed cause of 46,XX DSD is ovotesticular DSD (33%) followed by testicular DSD (27%). This is in contrast to other studies where CAH was found to be the most common cause of 46,XX DSD [20, 21, 28, 29]. Our findings may be explained by genetic differences and the low rate of consanguinity in our setting. Two out of the 3 cases that had a history of parental consanguinity had presumed 46,XX testicular DSD while 1 had 46,XX ovotesticular DSD, suggesting a familial/genetic rather than sporadic occurrence in these cases.

Despite the fact that a large proportion of patients were delivered in hospital, ambiguous genitalia was initially observed by the patient's mother in majority of the cases. In addition, 16 cases were never referred to the endocrine clinic. This may be because midwives and primary healthcare doctors are not trained on this, and there is no national guideline for diagnosis and management of DSD.

Ninety-five percent of our patients were identified at early age, thus “late” presenters may have been missed or diagnosed as late puberty. It is also possible that patients with for instance CAIS and 17*β* hydroxysteroid dehydrogenase deficiency with “normal” female genitalia at birth and later presentation of inguinal hernia with gonads may have been missed.

The rates of underweight and stunting were higher among these children than the national rates according to the Kenya Demographic and Health Survey of 2014 (11% and 26%, respectively). Apart from one child who was on management for severe malnutrition, it was not evident from the records whether these children were abandoned or neglected.

Eight (32%) out of 25 patients had laparoscopy/minilaparotomy findings that contradicted the abdominopelvic ultrasound findings. Ultrasound is operator-dependent. Therefore, when resources are limited, laparoscopy or minilaparotomy performed by an experienced surgeon may be a cost-effective way to identify internal structures.

While patients who underwent gender reassignment were more likely to undergo psychological evaluation, only half of them were documented to have been evaluated by a psychologist. In our setup, patients who need to change gender go through assessments by the psychologist, surgeon, and paediatric endocrinologist and, when required, have a meeting with the family and all team members. The family is then followed up by the psychologist. The Kenyan taskforce found that persons with DSD unanimously felt that surgery should be delayed until the child reaches puberty when the dominant sex characteristics have manifested, and the child should consent to the medical and surgical interventions and have all the necessary medical diagnostic tests done before the surgery [18]. A cautious approach to gender reassignment is recommended and should always be initiated by the patient to minimize gender dissatisfaction [30]. Skilled specialists should evaluate the patient taking into consideration biological and social factors, as well as sexual and fertility potentials [30]. Any psychiatric comorbid disorders should be addressed to improve compliance to hormonal treatment [30].

Ediati et al. [31] in a case-control study assessing emotional and behavioural problems in 118 late identified Indonesian patients with DSD aged 6 to 41 years found that patients who were untreated for most of their lives suffered more emotional and behavioural problems. Mean depression and anxiety scores have been found to be higher compared to population norms in males with Klinefelter syndrome and males with XY-DSD [32]. Self-esteem, body dissatisfaction, and experiences of shame were associated with psychiatric symptomatology in many DSD conditions [32]. It is, therefore, important that these patients receive timely education and counseling to improve their understanding and acceptance of the diagnosis. All parents of newborns with suspected DSD where there has been a delay in gender assignment and all adolescents with a newly diagnosed or existing DSD requiring medical or surgical attention should be offered clinical psychology input [14].

Thirty-two (45.1%) of the 71 cases in our study did not have a karyotype done, while 26 (36.6%) patients did not have an etiological diagnosis established. This finding is higher than that reported in other series [21, 26, 29]. This was due to financial constraints and loss to follow-up of patients. Investigation of DSD in Kenya is very expensive and out of reach for many patients. In addition, molecular genetic testing for DSD is currently not available locally, and sending samples out of the country would still require funding from individual patients since it is currently not financially supported by the government. Fifty-four percent of the respondents interviewed by the Kenyan taskforce described their experience in accessing healthcare as poor due to high cost of treatment and presence of very few specialized hospitals [18].

In conclusion, the commonest cause of DSD was ovotesticular DSD in contrast to western studies, which found CAH to be more common. There should be at least one multidisciplinary centre for DSD in Kenya, including a paediatric endocrinologist, psychologist, paediatric urologist or surgeon, and geneticist. There is a need to train geneticists to be included as members of the DSD team due to patients with karyotype anomalies, to interpret findings, and to provide genetic counseling for families. Psychological evaluation should be carried out for all patients at diagnosis and support given for families to improve understanding of diagnosis and avoid stigma.

Further studies should be carried out to identify the molecular genetic etiologies. Financial limitations make investigation and diagnosis of DSD difficult; hence, investigations should be supported by health authorities directly, through health insurance and increased budget allocation for research. A network for detailed diagnostics in resource-limited countries would be highly desirable.

There is a need to train health care workers/medical students for early diagnosis. A national guideline should be developed for diagnosis and management of DSD during and beyond the paediatric age group, and this should be re-evaluated and modified regularly according to emerging evidence and available resources. Every child with DSD should have a karyotype done.

## Figures and Tables

**Table 1:** Etiological classification among karyotyped patients (*n* = 39).

Main category	Etiologic diagnosis	N	Final gender assignment	Age at final gender assignment (years)
Sex chromosome DSD (*n* = 2)	45,XO/46,XY mixed gonadal dysgenesis	1	Male	2
	45,X Turner and variants	1	Unassigned	

46,XY DSD (*n* = 19)	Disorder of androgen synthesis/action	6	Male	0.2, 0.3 (2 patients), 0.5, 1.5, and 2
	Disorder of testosterone biosynthesis	4	Male	2, 4, 5, and 9
	5*α*-Reductase deficiency	2	Male	0.4 and 1.5
	Syndromic associations	2	Male	0.2 and 0.3
	Gonadal regression	1	Male	9
	Partial androgen insensitivity syndrome	1	Male	3
	Not established	3	2 male, 1 unassigned	0.2 and 0.3

46,XX DSD (*n* = 18)	Ovotesticular DSD	6	4 male	0.1, 0.8, 2, and 8
			1 female	12
	Testicular DSD	5	3 male, 2 unassigned	0.4, 1, and 3
	Congenital adrenal hyperplasia	3	Female	1, 4, and 7
	Ovarian dysgenesis (right)	1	Female	9
	Syndromic associations	1	Female	0.3
	Iatrogenic (non-CAH androgen excess in syndromic baby)	1	Female	0.1
	Not established	1	Female	2

**Table 2:** Etiological diagnosis (*n* = 71).

	Frequency (n)	Percent (%)
DSD etiology		
Ovotesticular DSD	10	14.1
Congenital adrenal hyperplasia	8	11.3
Disorder of androgen synthesis/action	6	8.5
Testicular DSD	5	7.1
Disorder of testosterone biosynthesis	4	5.6
Syndromic associations	3	4.2
5*α*-Reductase deficiency	2	2.8
Ovarian dysgenesis (right)	2	2.8
Gonadal regression	1	1.4
Iatrogenic (non-CAH androgen excess in syndromic baby)	1	1.4
Mixed gonadal dysgenesis (45,XO/46,XiYq (15))	1	1.4
Partial androgen insensitivity syndrome	1	1.4
Turner variant (48,XYYY/45,X)	1	1.4
Not established	26	36.6
Total	71	100.0

**Table 3:** Karyotyped and nonkaryotyped patients in terms of outcome.

	Karyotyped	Not karyotyped	*p* value
Patient outcome (*n* = 71)			
Still followed up	27 (69.2)	9 (28.1)	<0.001
Lost to follow-up	6 (15.4)	20 (62.5)	
Transferred back to referring hospital	6 (15.4)	2 (6.3)	
Inadequate documentation to determine outcome	0 (0.0)	1 (3.1)	

## Data Availability

The data used to support the findings of this study are restricted by the Kenyatta National Hospital-University of Nairobi Ethics Review Committee, and Gertrude's Children's Hospital Ethical Review Board in order to protect patient privacy. It may be released upon application to the ethical boards, who can be contacted at uonknh_erc@uonbi.ac.ke and info@gerties.org, respectively.
